# The Independence of the Soul

**Published:** 1860-07-01

**Authors:** J. L. C. Schroeder van der Kolk, William Daniel Moore

**Affiliations:** M.R.I.A., Honorary Member of the Swedish Society of Physicians and of the Norwegian Medical Society


### Art. III.—THE INDEPENDENCE OF THE SOUL:

### PROVED BY A CONTEMPLATION OF MAN IN HIS VARIOUS PERIODS OF DEVELOPMENT.

By J. L. C. SCHROEDER VAN DER KOLK.

(*Translated from the Original, in the "Album der Natuur,"** by William Daniel Moore, M.B., M.R.I.A., *Honorary Member of the Swedish Society of Physicians and of the Norwegian Medical Society.*)

When we look around us on the works of nature, and contemplate their infinite variety and richness, while all are brought in harmony and order to a system, nothing excites our amazement more than the universally diffused superfluity of life and motion in the organic kingdom, which, both in plants and animals on the whole maintains its standard in the midst of incessant change, of perishing and starting into existence, unless we discover the concealed Artificer, who directs and sustains it all.

But if we direct our attention to a single living being, and endeavour to discover the connexion between the operations of life and their causes, we find no less order and harmony, and are brought to the conviction that throughout the whole creation means and object coincide ; that in it every part exists, and works not for itself alone, but also for the existence and life of the entire body ; that all is arranged in inscrutable wisdom, that every plant and every animal is formed, that their internal actions and powers are regulated, that their endowments and properties co-operate in harmonic order, precisely as their being, mode of life, and necessities demand; that nothing is forgotten, nothing is useless or superfluous, but everything proclaims the Almighty Maker, whose perfection is reflected in his works as in a mirror.

But if we contemplate man himself, we discover, in addition to the operations of his body and of the nervous powers which govern his corporeal life, still other new and higher capacities and endowments, which we meet with nowhere else in the same mode in Nature around us. We here observe our *higher I*, our spirit endowed with reason and understanding, capable of tracing and investigating the wonders of Nature, of estimating cause and

* Part V. 1852.

effect, of raising us to the Supreme Cause, to the Creator himself, and of honouring him as the Infinite Wisdom and source of all.

Not only the nature and essence of this higher principle, but also the connexion which unites the soul so closely with the body, has at all times been a question which men have in vain endeavoured to solve. Pretty generally we represent our soul as a higher independent principle, of which our body is only the temporary abode and organ; but many, particularly in our day, regard the soul only as an emanation of the powers peculiar to the living body, and connected with matter, or as a manifestation of power and action produced by metamorphosis of tissue in the nerves and brain; according to Ludwig Fick, of Marburg, as an union of central nervous currents,* but to which, according to him, as a product of bodily forces all independence must be denied, and which is thus completely *one* with the body, *one* with matter, in whose action it is stated to originate, and as frail and perishable as the acting forms of matter, to which it is indebted for its appearance.

That, as in the other works of creation, soul and body co-operate in harmonic connexion to a common object, cannot be doubted; that the influence of the body on the mental powers and on our higher being is exceedingly great, daily experience teaches, our own constitution, temperament, our more or less violent inclinations and passions prove, and the phenomena of insanity demonstrate to us with melancholy certainty.

But do in fact Nature and all these phenomena teach us, that soul and body are so completely one, and that our higher *I* is only the product and the expression of our highest bodily powers, sprung from the metamorphosis of tissue? Or does an attentive consideration rather show us that the soul is not so entirely the immediate product of the body, but that, on the contrary, the body is the organ of the soul, which as an independent being, whose nature we cannot here penetrate, dwells in the body, and only through its help can here below attain its full development?

Important questions certainly, with which our tenderest and dearest interests are so closely connected; questions which at all times have constituted the great stumbling-block to philosophers and sceptics.

The importance of the matter will surely plead my excuse to the readers of this Album, when I endeavour to ascertain, by opening the book of Nature itself, whether we cannot in it find some elucidation and solution of these weighty though obscure inquiries. With this object I shall, simply following the footsteps of Nature, briefly sketch the whole man in his successive stages of development: the opening of his higher mental powers

* Müller's *Archiv*, 1851, Heft V., pp. 385, *et seq.*

in the child, their further formation in the youth, their full vigour in manhood, and lastly their maturity in the period of old age,—in order so to examine, whether Nature in fact teaches us that our higher *I*, our intellectual capacities and endowments, our reason and moral feeling are only effects of bodily forces, and therefore keep such equal pace with bodily actions in the several periods of life, that we may assume the perfect unity of soul and body.

---

Immediately the newly-born child has entered the world, he is aroused from his hitherto undisturbed slumber, in which he could receive scarcely any stimuli from without, by new sensations never before experienced by him. His senses do not, however, yet possess perfect capability of correctly transmitting these sensations, and his as yet undeveloped mental powers are still unable properly to receive and distinguish them; they are only impressions and sensations, there are as yet no perceptions.

The first life of his soul consists only in the transient reception of impressions which he does not yet comprehend; he enters his first school to learn to take in and distinguish the sensations he receives, and thus by frequent repetition to acquire the power of recognising and understanding them; the impressions on his senses lead to perceptions; they are, as it were, the mental food the world offers him, the first material for his thought. Besides the new impression of light, which meets his eyes, he seems scarcely to experience any other perception than that of the to him strange feelings of hunger and thirst. Previously constantly fed, he is now quickly aroused from the beneficial sleep wherein he, as it were, continues his fœtal life, by the first unpleasant feeling of hunger or thirst, extorting the involuntary cry; his own voice, which he now unlooses, is itself among his first perceptions. But for all this beneficent Nature has provided; the movements necessary to the act of suckling are not directed by the will or understanding, but are at first involuntary; so soon as anything touches his sensitive lips, this sucking movement spontaneously commences, and even children born without a brain, perfectly perform the act of sucking. So long as the child is still unable to govern his own body, this guidance is undertaken by a peculiar artificial arrangement of the system; all is cared for, nor is anything left to his inexperience and as yet undeveloped will and power of acting.

On the tender, warm bosom of his mother his first necessity is supplied, and he there receives the agreeable sensation of satisfaction and content; it is the first enjoyment of life which the new world offers him. The constant repetition of this want, with the succeeding enjoyment of satisfaction makes this sensation more lasting and persistent; very soon when he is taken up, or perceives any strange sensation, he draws his little mouth aside anew to satisfy his want and find his enjoyment; for as yet he does not distinguish his mother's breast from any other novel stimulus or perception, and thus in his still obscure consciousness he makes the first advance towards a higher development; awakes the first trace of memory, which begins to give him a misty feeling of a previously tasted enjoyment; he already commences to live in the past.

His senses are, however, still imperfect, and only gradually does he become capable of further impressions; at first interrupted by the constant need of sleep, the stimuli of the senses are administered to him in small, frequently repeated doses, and thus he is preserved from over-stimulation.

At first he seems deaf, or at least hard of hearing; the cavity of the tympanum is still filled with fluid, which seems to disappear but slowly, and to make way for the impressing air; this existence of fluid in the cavity of the tympanum must make him deaf, as not unfrequently occurs also in after life. But I have often observed distinct evidence of hearing within a few weeks; although the child is at first not nearly so easily disturbed in his tranquil sleep by a great noise, as is subsequently the case.

Sight, our highest sense, gives him his first perceptions, and brings him into closer relation to the outer world. I have seen a child, even a few hours after birth, follow with his eyes the movements of a candle at some distance, in which respect he is immediately distinguished, according to Burdach, whose accurate observations I here chiefly follow, from young animals, who are stated not to do this.* But the convexity of his eyes, and the lenses contained in them, seems still for a rather long time to limit his vision more to near objects, and the immobility of the globe of the eye and the membrane frequently spread over it, seem somewhat to obscure his vision; he rejoices in the light, but does not yet see, that is, he does not yet perceive.

He first follows the light, afterwards lighted objects and their movements, and soon these repeated impressions begin to excite a peculiar activity of the mind, which, as if hereby aroused from sleep, commences to manifest its peculiar action; light already makes an agreeable impression on him, and soon he appears impatient of being in the dark; by constant repetition be begins during the early months to attain to a certain recognition of objects; what is new seems to give him some pleasure, and the first involuntary smile around his tender mouth in the second or third month puts his watching mother in a transport at the rapid development of her darling. In the third month he begins distinctly to express pleasure or dissatisfaction. At the same time,

* Burdach, *Die Physiologie als Erfahrungs-Wissenschaft* III Th. p. 185.

memory and the capability of combining impressions increase. Formerly when he felt hungry he let his voice be heard until, applied to the breast, he found satisfaction in the act of sucking; in the third month he generally becomes quiet when he is taken up to suck; he knows now, by repeated experience, that his wants will soon be supplied; a little later he discovers the effects of crying, and now cries designedly in order to obtain something. Thus a peculiar activity of the spirit begins to be developed, his memory becomes stronger and he makes his will known. In fact, a remarkable phenomenon; let us consider it for a few moments. It is said: Soul and body are one, or the soul is nothing else than brain or nerve-power; but does Nature indicate this to us, when we observe her without prejudice? We know no nerve or brain-part, which of itself acts alone and definitely in the same manner reflects a received impression. Here we see a new principle, an independently acting essence or power gradually developing itself as it were out of sleep, a principle which begins to manifest volition and consciousness, whereof we discover no trace in any single nerve-force,—a being which acts on brain and nerve-force, or receives and takes in impressions, which guards, acts, appropriates, and gives away again, but does not, as in a mirror, immediately reflect; on the contrary, it acts according to its own will; according to a peculiar independent power, and is no longer merely passively driven. I cannot in fact read in Nature this similarity and identity between soul and body and their mode of action, but a peculiar independent principle, which must be still further developed.

As in the first period the child's spirit is passive in the reception of impressions, without as yet manifesting any peculiar activity, so it does not yet act upon his body; the first movements are involuntary and undefined; he is still without the idea of touching anything, nor does he guide the movement of his arms. But at a very early period he can bring his little hands to his mouth; subsequently, in the third month, he catches at an object to endeavour to raise it to himself; proper touching and handling succeed much later, and demand a higher degree of mental activity, and special investigation. Hence the absurd opinion of some writers, who assert that the child receives the first impression of distance and size by the touch, and by feeling learns to see. On the contrary, he sees and distinguishes objects at various distances long before he seizes them with his hands and begins to examine them; he is not yet capable of the philosophical speculations and deductions which these writers in their fancy ascribe to him, imagining a child in whom a little philosopher should be hidden, already reasoning and drawing conclusions as to the properties of things.

At the end of the third month his development very rapidly increases, his attention becomes more acute, he already endeavours to imitate, and at this period I have even seen him accommodate his mouth to counterfeit a sound he heard; a rapid change of objects surprises him, and he crows with delight. But even now a new phenomenon is developed, the first swellings of passions appear, against which he will hereafter have to contend so much; he makes his displeasure and anger plainly known, he cries and plunges with his legs, and resists as much as he can, when he is being washed; by the different tones of his cry he already expresses what is passing within him. Correctly does Burdach observe, "No animal is after birth so impatient and passionate as man, because man alone is endowed with an independent spirit which endeavours to break through restraints and obstructions."

Simultaneously with these passions his mental affections and his feeling are developed; in the commencement he is passive and as yet incapable of joy; this sentiment must first be excited by repeated agreeable impressions; at first they are only impressions on the senses, such as those produced by brilliant objects, which procure him pleasure; soon the gentle human voice produces this effect; and in the fourth month he crows with delight when he is spoken to or sees a friendly face. The repetition of this renders the enjoyment of pleasure a necessity; he becomes sociable and does not wish to be alone; habit—so well called by Burdach a recollection of feeling—begins to exercise its power, and with it education commences. By daily habit he becomes attached first to his mother, with whom he finds rest and satisfaction, and subsequently also to the other members of the family. The desire for companionship thus excited is the first germ of returned love, and thus is developed the noblest of human affections, love; first towards his mother, afterwards towards his sisters and brothers, and farther, as the circle of his acquaintance extends, towards other individuals. This feeling is excited chiefly by hearing and thus by being spoken to; how much hearing acts on sentiment is shown by those born deaf and dumb, who are in general much less sociable and more capricious; they have much greater difficulty in restraining their passions than those have who are born blind; thus the sound of lamentation moves us much more than the sight of an unfortunate: sound acts more on the sentiment and speaks to the mind, sight acts more on the understanding.

As he advances, the child begins also more rapidly to distinguish what is strange and unusual from what is already known; he first stares at a stranger with wide-stretched eyes, then turns away his face, hides himself in his mother's bosom, and begins to cry; a new affection, fear, is manifested, and the child becomes uneasy and shy on the approach of an unknown person.

In like manner he begins to recognise what appears pleasant and remarkable; he desires to grasp it, and in the desire to seize it the first love of property is developed; he is still quite an egotist, the idea that anything can belong to another he acquires much later, and only by sacrifice and loss; and if it promotes the acquisition of what he desires, a peculiar restraint over himself. No wonder that this is difficult to the child, as we see that in this respect so many men continue children all their life through.

If his desires are constantly satisfied, if he observes that his wishes are officiously complied with, and that he gets what he demands, he learns more and more the power of his will, and obtains by crying what he cannot directly take by force. If he is not always attended to, and if something is withheld from him, he experiences the law of necessity, is obliged to restrain his desires, subjects himself to order, and learns to obey. On the contrary, by too quickly complying with his wishes, he is rendered the victim of imperious desire; by finally yielding to him, his capriciousness is nourished, and the power of governing himself, the highest power in man, is not acquired by him; his higher development is retarded, he becomes capricious and obstinate; he continues a child, and is completely spoiled for the whole of his subsequent life, if opposition and the force of circumstances do not at a later period bend his stubborn will.

With each succeeding month the child rapidly advances in the development of his mental powers; his memory in particular becomes more acute; he recognises with delight objects seen before, and soon recollection of things he no longer sees ensues; he acquires the power of bringing them in his ideas before his mind, and of, as it were, delineating them in his thoughts, his imagination awakes, and even now manifests itself in his dreams.

If he has in the fifth and sixth months learned to seize objects, he commences to busy himself with them, his mind exhibits more special activity, he begins to play and to investigate. He already more and more makes his desires known by definite sounds, and in the eighth month distinctly endeavours to imitate sounds and words; he for the first time tries to express not only his wishes, but also his ideas by the use of language, exhibiting a capacity for it designedly conferred on him by nature, and which at a later period of life appears to us nearly incomprehensible.

We must, however, here observe, that the child understands the meaning of many words, and, for example, recognises his name and that of his parents before he can pronounce them. If any one hears a wholly unknown language spoken, this kind of acquisition is not easy; we require an interpreter, a teacher, a grammar, and a dictionary; but the child learns to speak without this aid; he has neither dictionary nor interpreter, and although some names by constant repetition become recognisable to him, these are perpetually brought before him in varied senses. How much attention is required to understand the often figurative signification of adjectives—for example, the sweet child, a sweet lump of sugar; fine weather, a fine garment, a fine doll; how much observation to understand the verbs, which represent no visible thing, in the several conjugations and meanings in which they occur, and to distinguish them in a quite different order and connexion which he does not comprehend; how much to comprehend the meaning of colours and of numbers; and still examples are not wanting of children, educated by French nurses, learning two languages at once. Indeed, a friend of mine informed me that he knew a child born of Dutch parents, at Verviers, who at the age of four years made use, according to circumstances, of four different languages without confounding them, namely, of Dutch, French (the language of most of the respectable inhabitants there), Wallonic (the ordinary dialect of the lower classes), and German, the language of some families living at Verviers, with whom his parents were acquainted.

The child, in fact, in this respect, exhibits a surprising capacity of mind, which at a later period of life we do not possess, and which elevates him far above all animals, as the parrot learns indeed to imitate particular words, but does not understand their meaning. We can teach the child only the names of objects and persons; abstract ideas and special properties, which are not the object itself, are learned only through the peculiar operation of the child's mind, and without any deliberate method.

In this we see particularly the fitness of our body to nourish the mind, not only by conveying to us impressions on our senses, sounds, and words, but also by the power it gives us of reflecting our thoughts in sounds and words as speech and language. It is specially thus that the endowments of our mind are developed. Precisely through speech and the signification of words is the child's attention more forcibly directed to surrounding objects, and he becomes acquainted with their properties. Words and names are the marks for our memory, and the name recals the thing itself. Numbers the child learns last, and with the greatest difficulty, just like many savage nations, who do not carry them beyond a low figure. But if we try, as Gerdy correctly remarks,* to count the number, for example, of writers in our library without thinking of figures, by repeating the names alone, we do not reach half a score of books before we are in confusion. Thus it

* *Annales Psychologiques*, Tome 1, p. 374.

is particularly by the faculty of speech that man acquires beyond animals the power of developing his already much higher organization and understanding; it is by the assistance and means of the body that the understanding is cultivated; but are therefore our mental powers and thoughts actions of matter and developments of bodily power, or are they the actions of a special independent faculty, a peculiar principle, to the development of which the body must be subservient? In other words is our mind as in animals for the body, or is the perishable body for the mind, and only its temporary servant, through whose aid the mind may be developed? The answer to these questions will, as I hope, just now appear plain to us.

Speech, that excellent possession of man, is, as Burdach correctly observes,* not merely a result of the structure of his body and of the vocal apparatus. Many animals can imitate and articulate words without being therefore capable of speech, and the dumb invent for themselves, instead of speech, a language of gesticulation such as no animal possesses. It depends on man's power of generalizing phenomena in his ideas, and on the endeavour to reflect his ideas in a sensible form, so that by the mode in which such forms or signs are connected one with another, each thought may be expressed. Language is not given directly by nature, for each people has a different one; but is discovered by the proper action of the mind, only the impulse to it is innate; in fact the child would, if he heard no language in the society of others, create a special language for himself. This the deaf-mutes prove, and even those who are born blind and deaf and dumb learn to speak by feeling and attain a certain development, notwithstanding that their mind is shut out from by far the greater number of impressions on their senses. So little are the mind and spirit the result of impressions on the senses, so strongly do they on the contrary indicate the existence of a peculiar independent principle dwelling in the body, that I cannot refrain from quoting the following touching proof, communicated by Burdach, with very many others,† of blind deaf-mutes. Laura Bridgman in North America, became perfectly developed in acuteness of mental power and tender feeling, notwithstanding that she was blind and deaf and dumb, that her sense of smelling was wanting, and her taste so defective that she commonly mistook infusion of rhubarb for tea. She was admitted into the Blind Institution in Boston in 1837, in her eighth year; she soon felt happy there and was penetrated with thankfulness to her teachers, as in this institution she found more food for her mind than in her parents' house, at Hanover, in North America. After she had spent half a year in

* *Blicke ins Leben*, II B., p. 189.

† *Ibid.* III B., p. 53.

the institution she received a visit from her mother, felt her hands and clothes without recognising her, and thereupon turned from her as from a stranger; for the many objects and impressions which since she left her parents' house had attracted her entire attention, had in her limited powers of sense weakened the recollection of her home. She was delighted to get a string of pearls she had formerly worn, and she gave Dr. Howe, the director of the institution, to understand, that this was a present from her former abode; nevertheless she repulsed her mother, who wished to caress her, and returned to her playmates. On receiving from her mother another object from home, she became very much excited, examined it accurately, and informed Dr. Howe that this lady must certainly have come from Hanover: she also allowed her to caress her, but then again left her with indifference. After some moments, when her mother who was hurt again approached her, she appeared to be struck by the thought that this could be no stranger; she felt her hands very eagerly, grew quite pale and again as red as fire; hope and doubt were contending within her. Her mother drew her towards her and kissed her; upon which she threw herself on her bosom with an expression of transport, and left her no more. Playmates and playthings had no longer any attraction for her. On subsequent separation from her mother, the girl, now nine years old, showed as much understanding and consideration as deep feeling; she accompanied her, when leaving, to the front of the house, where she clung closely to her; then felt round her to ascertain who was near her. Observing a much beloved teacher, she seized him with one hand; while with the other she held her mother spasmodically; let go the latter, turned round, and clung sobbing to the teacher.

Does this touching ebullition of feeling and love, this action of the understanding, to which so few sensual impressions had access, express nothing more than a simple material operation proceeding from metamorphosis of tissue? or does it not rather indicate a peculiar independent essence, which, notwithstanding its much more defective organs of sense than many animals possess, elevated itself above all obstacles and independently and freely developed itself?

It is not by constant repetition of sensual impressions that our organs become more acute, we perceive them scarcely more at the last, but only by the proper independent attention of the mind to these or those perceptions, whereby we learn to observe more particularly; a person born blind has much more acute feeling, but after recovering his sight he gradually loses the finer sense of touch, as his attention is now distracted from feeling to vision. It is therefore the proper independent action of the mind, and not that of the organ, which gives us the capacity of finer perception, and must not the mind itself be an independent entity? The blind deaf-mute James Mitchell, in Scotland, came to know not only his house but even the country about it, went to walk alone, and returned home at the proper time, although he had only the sense of touch to guide him.* Burdach adduces a number of striking examples of the development of such persons and the mode of teaching letters and their significations through feeling alone, and so of communicating a language by the touch, as a proof that man may be developed in the absence of his organs, and demonstrate the independence of his mind. Much of this I might quote, did not the extent of the subject oblige me to abridge my remarks.

Simultaneously with the development of the mind, the child's body now increases in stature and strength. He learns to guide his movements, to grasp, to stand and finally to walk and move without support. By these daily exercises the body is strengthened, and its increasing power is reflected on the vivacity and activity of the mind and promotes the development of each.

In judging of others the child, in his still trifling experience, contemplates every thing from his limited childish point of view with reference to himself. Thus I have often seen a child in his third and even in his fourth year when reproved shut his eyes, with the idea that he could not then be seen; or with closed eyes catch at a forbidden dish, thinking that as he did not see, others could not observe his little epicurism.

But the nursery has already detained me too long, that important theatre, where man commences his education, and where so many seeds are sown and bud, which shall subsequently bring forth roses or thorns.

In his further development vivacity and mobility are the peculiar features of the child; he acts quickly in everything, both in his movements, thoughts, and ideas. Many impressions are also easily lost; to take root and to have a permanent effect admonitions must be frequently repeated.

The constantly renewed and always more perfect perception of objects which he acquires on all sides, his need of occupation, the capacity for impressions, in consequence of which every thing arrests him, make him inquisitive, and at length greedy of knowledge, his learning-time commences, and with it a peculiar activity of mind, which is directed less by accidental external circumstances and impressions than by his own will and inclination.

Thus he grows and becomes, from a child, a boy, and at length a youth; in no animal has Nature extended youth and learning-time to such a length as in man, for he alone must learn everything, and prepare himself for higher education. In this the difference of the sexes is soon manifested, in the wilder sports of

* Burdach, *loc. cit.*, p. 36.

the boy, who longs to exercise his bodily powers and independence, and with respect to his mind penetrates more deeply into the matters which come before him; while the more gentle girl, good and beautiful, outstrips him in general development, in tact and sense of truth. But on this subject time forbids me to dwell. It may suffice to indicate how large a part the body takes in the entire development of the mind and of the disposition. Even in the child and boy the disposition, indeed the whole character, exhibits itself, and becomes more strongly developed in the youth. The difference of frame gives to each individual the tendency and hue which subsequently pass into the temperament peculiar to each, so that in the same family each child manifests his own nature and disposition. Childless people, without experience, may argue very wisely on this subject, and often think that the newly born child is a white, unwritten-on sheet of paper, on which the parents may as they please inscribe what seems best to them. Experience shows that the paper is already fully written on by Nature, and we may think ourselves fortunate, if we can improve the sense, and place here a comma, there a semicolon, and above all, if we can introduce a full stop in the right place. The soul may indeed originally be one and the same; but the eye and the body are the spectacles through which each one observes with his own colours under different degrees of magnifying power and accuracy all around him; or the body is a peculiarly tuned musical instrument which more or less acutely conveys the impressions of the outer world with these or those particular notes, influencing the tone of the disposition. It is the body through whose aid the mind is not alone formed, but also, according to the constitution of each, receives a peculiar modification, which again changes with the period of life. But the body and education are not the sole sources of influence; even in the terribly neglected Caspar Hauser a very good disposition was subsequently developed. A child may be very much spoiled by bad education, but Nature has not left this altogether to the caprice of the parents. The child is not a piece of clay, out of which the parents can at will form a man or a wild beast. "The most noble principle," says Burdach, "the imagination, the elevation of the soul, the glow of moral feeling and love, are not learnt, but promoted."

This influence of the body we see also strongly marked in the youth, where the body more and more approaches to its full formation, where the muscular system has been developed, and the blood is driven forcibly through the vessels, and where also the mind unites vivacity with power, courage, and enterprise. With modification of the former fugitive nature of impressions, self-consciousness and reflection awake in him. He wishes to form himself by his own power, his learning as a child passes into study, inquisitiveness into love of knowledge, and empiricism into science. He strives after wisdom and self-formation, and while he wishes to act independently abroad, his parent's house becomes too narrow for him.

But quickly in the already sedate youth the current of the blood excites him in his fermenting vivacity and passion, and he loses the control over his affections; they overpower his mind, he is dragged along and now returns in his passion to the condition of the child, which cannot guide itself. At the same time the bodily operations are exalted, the current of the blood is more rapid, the metamorphosis of tissue is more active; but does he now in consequence become wiser? Is his judgment at that moment more correct—his moral feeling exalted? Is he not like an insane person, in whom, with still stronger corporeal impressions, the mind is wholly carried away by the storm of the feelings, but whose subsequent recovery shows that it was not thereby altered, and that it lost nothing, but has preserved its peculiar powers and capacities? Does it not prove in a peculiar manner the action of the body, and the desires springing from it on the mind, that among the insane many think themselves higher, and imagine that they are princes, kings, or emperors, and that they can control millions? Others believe themselves bad, criminal, or forsaken of God. But I have never seen an insane person who thought himself more virtuous, braver, or more philanthropic than another.

But if the brave youth has through severe hemorrhage or illness lost his strength, his courage and gaiety and his enterprise have disappeared, but his understanding is not lost—his moral feeling is not extinguished. Does not nature thus distinctly show that the soul is a peculiar independent essence, although connected with the body, not wholly bound up with it nor perishing with it?

In the powerful constitution of the youth, however, new sensations bud, living, strong impressions, and the storms of passions and inclinations besiege his mind. It is the most important, but at the same time the most dangerous period of life; it is the strife for dominion between body and soul; it is the conflict on which it depends what he shall be, whether he shall overcome himself and his desires, and by his own power learn to stand firm as a man, or shall yield to his impressions, desires, and inclinations, and by giving way to his passions return to the minor condition of the child, and perish as a drunkard, voluptuary, or covetous criminal. Fortunately in this emergency a gentle genius comes to his side, who can guide him through all the tortuosities of life, and who, though he may for a time turn a deaf ear to its voice, never entirely forsakes him. This is the voice of conscience, peculiar to man alone; it is the feeling of duty, right, virtue, and piety, which in this contest offers him the palm of victory. This is not an acquired knowledge; even without being instructed in it by man, a deaf mute knows, and even a blind deaf mute by his innate feeling, what is good and what is evil, what is right and what is wrong.*

Formerly as a child a complete egotist, the desire of acting buds in the awakened feeling of the vigorous youth; but not exclusively for his own honour and glory, he desires also to live for others; his heart must learn to beat strongly for all that is great, and good, and beautiful. What is transitory and fugitive no longer satisfies him; he has not enough in himself, love kindles in his mind, and his fancy holds up to his eyes in her mirror an imaginary world, but the reality is still strange to him. Burdach says of him,† "The unity of life and the contentment of childhood have departed from the youth, and he feels with sorrow that ripening individuality does not bring him the happiness which, as a boy, he had expected; he is overcome by an undefined desire, an imperfect feeling, and dissatisfied he turns his glance from the present to the future, from the real to the imaginary." Thus he lives in part in the future, which his lively fancy clothes in the brightest colours; it is his season of poetry.

And thus he at length emerges from his realm of dreams and imagination into the rude reality of the world. This, however, frequently does not take place without many blows and disappointed expectations, but while he thence learns the vanity and exaggeration of many of his ideas, the hard reality of experience and truth often forms him into a man.

Schiller strikingly describes the youth in his bold expectations and courage, in his *Die Ideale*:—

Wie sprang, von kühnem Muth beflügelt

Beglückt in seines Traumes Wahn,

Von keiner Sorge noch gezügelt,

Der Jüngling in des Lebens Bahn!

Bis an des Aethers bleichste Sterne

Erhob ihn der Entwürfe Flug;

Nichts war so hoch und nichts so ferne,

Wohin ihr Flügel ihn nicht trug.

But not less strikingly his disappointment—

Es dehnte mit allmächt'gem Streben
Die enge Brust ein kreisend All,
Herauszutreten in das Leben,
In That und Wort, in Bild und Schall.

* Burdach, *Blicke ins Leben*, p. 46.

† *Phys.* l. c. page 291.

Wie gross war diese Welt gestaltet,
So lang die Knospe sie noch barg;
Wie wenig, ach! hat sich entfaltet.
Dies wenige, wie klein and karg!

In this sometimes hard conflict, his system, becoming with his time of life more and more sedate, comes to his aid; his strength he has still retained, and it is even increased; his mental powers are not blunted, but his blood no longer circulates so rapidly and foamingly through its vessels; his less impetuous constitution renders him more proof against shocks, and no longer sweeps him along so irresistibly in passion. His less stimulated brain, the organ of his mind, makes him adapted for more composed and calm reflection; his imagination, already purified by experience, no longer soars so high; he listens more to the voice of reason, considers more clearly, and having by experience learned to distinguish between what is real and what is only apparent, he becomes more attentive to the connexion between cause and effect, and calculates more deliberately and with more precision the results of his acts; he is better able to govern himself, his understanding and reason obtain preponderance over his organism, he becomes more independent of himself, and learns to stand as a man amidst the storms of life.

If he thus appears in this conflict, on this great crossway of life, like another Hercules, as a successful conqueror, he will stand as a man in the equilibrium of his full powers; formed by education, by his matured understanding, reason, and awakened moral and religious feeling, and instructed by experience in the reality of life, he has acquired the power of mastering himself and has thus become ripe for social freedom; he is human, he is a man, for maturity as a man necessarily includes the power of governing himself.

His former fancies and dreams have not indeed been fully realized, but in his station as an active and useful citizen of the State, as a loving spouse and father, his aspirations are fulfilled; and in his efforts for the public good and for his household, he finds his peace and enjoyment. Previously, rather an egotist and living for himself, he lives now for others, and finds his happiness in theirs; and this pure enjoyment procures him much more genuine and higher happiness and satisfaction than the undefined stirrings and wishes of youth with all their rosy colours could afford. Forcibly and truly does Tiedge express this—

Durchschaut das ganze Lustgebiet:
Kein Paradies für Engel!
Was diese Erd' einmal erzieht,
Hat auch der Erde Mängel.

Nur *eine* Freud' ist unbefleckt;
Und diese Seelenweide,
Die schon nach Himmels Wonne schmeckt,
Heisst Freud' an fremder Freude.

This is with man the period of action; and though all cares may trouble him, they are stimuli which lead him, by perseverance, to overcome the troubles of life. By abundant intercourse with men he learns, often as he may stumble therein, to judge each more from his own point of view; it is to him the reality of life, he distinguishes the true from the apparent.

But we must again ask, does nature in this change of bodily and spiritual condition in mature age, teach us that soul and body are one? Does she show that the soul is an emanation of the corporeal powers because the more composed system co-operates harmoniously, whereby the mind in its more calm and sedate reflexion and action is now less tossed about and acquires the mastery over the affections of the body?

Certainly not! But as everything in nature works for an end, and is adapted to its object, so the more composed constitution gives to the maturer period of life the calmness and the power to guide the reins of the understanding. Napoleon's pulse was usually only forty beats in the minute, or little more than half that of an ordinary man, and this circumstance certainly contributed very much to the maintenance of his calmness and composure in the most important moments of his stormy life; but who will, on account of his slow circulation, deny to Napoleon clearness of mind and powerful, rapid action of the soul?

The brain of an adult man is not to be distinguished, either by the knife of the anatomist or by the most careful microscopic examination, from that of a youth or even of a boy, and yet what a difference in the mind! If this proves that mind is cerebral force, why, I ask again, is the perfect understanding of mature age not present with the more lively metamorphosis of tissue and action in the brain of a boy? Does not nature, on the contrary, show us in all this, that our mind is an independent separate principle, a special power which is indeed developed through the medium of the body, and strives after perfection, but is not therefore one with the body?

But I should fear to be tedious, did I dwell longer on this point. Besides, I think what has been said is sufficient for our present object. I shall now pass to the last division of my subject—namely, the consideration of the period of old age.

In general, we do old age an injustice when we represent it under the image of a decrepit, dull, deaf, and cold individual. It is true old age has its faults, many of which are, however, the evil fruits of early life; but we need not, therefore, borrow the image of an old man from ill-health, any more than we need represent youth by a consumptive stripling, because that disease belongs more peculiarly to youth ; we speak of a sound old man, and ask, what changes in the constitution give the tone to his mind and disposition ? Burdach says correctly :—" Life is in its essence from the commencement to the end an harmonious expression of forces, of which the one is therefore a counterpoise to the other, and a natural normal disease is a nonentity. Thus, as old age is not in itself marasmus or wasting, so neither is it dulness of intellect nor dementia."

On the contrary, what some represent as a defect of old age, is a wise and harmonious arrangement. The principal character of the old man is, that he is more turned in upon himself, is less affected by the outer world, and acts less outwardly. I shall endeavour to point out the intention and beauty of this arrangement.

The changes which have taken place in his body contribute much, indeed everything, to distinguish the aged in his actions from the strong man. The old man no longer possesses either the vivacity of youth or the strength of the man ; he is no longer so deeply affected by what daily passes around him, and his intercourse with the outer world has become less active ; he is more turned in upon himself ; but all this is a natural result of the changes which have taken place in his body. His senses are duller, his muscles have become weaker, consequently the impressions he receives from the same are blunted, and his external force and action are diminished ; he no longer participates in the lively bustle of youth which wearies him, and to which he is now unequal ; the inclination to repose and rest is the natural effect of his present condition, and this increases in him.

But as his circulation is retarded, and his heart beats less powerfully and actively, while his nerves are blunted and respond more slowly and less vehemently to impressions, he becomes also less excited by passions ; his desires are, as Cicero so excellently describes in his *Cato*, more moderate, he is less eager and less passionate, and with this diminished vivacity of his constitution and fancy, calm deliberate reason and correct judgment, matured by long experience, have acquired the preponderance. He has learned the true value of things in this changing life, and is no longer carried away by fickle false enjoyment In consequence of the diminished impressions from without and the lessened acuteness of his senses, present and daily occurrences receive less of his attention and he becomes more forgetful ; his memory for the passing course of events becomes weaker.

But it is very remarkable that the recollection of his earlier days, of his youth, of what he has done and acted as a man, remains before his mind with unextinguishable clearness. It has become the property of his soul, he lives in the memory of the past; Nature allows him to retain the fruits of his experience, that he may be enabled to judge correctly of the value of things. Hence he rarely undertakes what is new, of which he knows not whether he shall attain the end, but having reached the autumn of his life, he gathers, like an husbandman, the fruits of his labour.

But with the collapse of his body, with the retardation of his circulation and the diminution of his strength or blunting of his nerves, his understanding is not necessarily impaired. On the contrary, a clearer mind is often concealed beneath the silver hairs, and wisdom and correct judgment have at all times been attributed to old age. "We should be very much deceived," observes Professor Pruys van der Hoeven,* very forcibly, "did we imagine that behind wrinkles and beneath hoary locks the cold and frost of winter reign; in the inner man the fire glows which once flamed forth externally." His higher *I* does not succumb because its dwelling has becomes stiff and fragile, but just as his eye is far-sighted and less capable of observing in detail adjoining small objects, he reviews more clearly, like a Humboldt in his *Kosmos*, the great, the universal, and the distant, and often hands over to his posterity or his friends and relatives, at the command of truth, right, morality, and piety, the matured fruits of his life and experience. Thus by his counsel he is still useful to others, although less active in society; and although by constitution less excitable, he has not therefore become insensible to the welfare or sufferings of others. A short time ago I heard the venerable Maurits Cornelis van Hall, aged eighty-four, recite an excellent poem in touching accents at the sight of the benefits conferred on so many unhappy individuals in the institution for the insane at Meerenberg.

By experience acquainted with the transitory and changing nature of most things, the old man holds more firmly to that which he has found to be permanent and lasting; hence the feelings of truth, duty, virtue, and piety, occupy the foreground, especially among the aged. "Nowhere," says Rush very strikingly,† "do we find an instance of moral qualities or religious feeling by which a man was distinguished, being weakened in old age." This, however, is generally admitted: if we excuse faults and unsteadiness, although we disapprove of them, in youth, censure and dispraise them in the man, in the aged they excite abhorrence and contempt. The old man, although he still participates in the suitable cheerfulness of his friends, has at the same time become

* *Anthropologisch onderzoek,* 1851, page 196.

† Burdach, *Phys.* Th. III., page 426.

more serious, and turned in upon himself; his children, now adult and independent, have for the most part left their parents' house; the young people, in the greater activity of their dealings and pursuits, spontaneously separate from the aged, and follow their own inclinations; the old man's former companions and friends have mostly gone before him, and the later generation, having grown up with other impressions and views, no longer sympathizes with him. Thus he becomes more left to himself, and lives, in his unweakened memory of earlier days, rather in the past and in the future. As man he has fulfilled his duties towards society and his family; he has lived for others; having approached the end of his career, he lives more for himself, and his spirit, in reflecting on the past, reaches forward towards his future fatherland. And thus by his constitution and circumstances—yea, even by nature herself, he is led to collect from his former life, for his own final development, the lessons of experience, of wisdom, and their fruits; the unimpaired memory of his early days holds up his own life before his eyes as in a mirror; and by the prospect he is led at his approaching end to self-examination. He takes account with his life.

What he in a well-employed life formerly endeavoured laboriously and earnestly to attain to, has in fact become his, his passions are subdued, the heat of the conflict is over, and the peace of the conqueror is his reward. Reflecting on his former life he becomes spontaneously filled with gratitude towards the Author of all good, who has thus far crowned him with so many benefits; the thought of his approaching end exalts his religious feeling; and in the conviction that the inner voice, which never wholly forsook him, is that of truth, he looks forward with calmness and tranquillity to the future, which he awaits with confidence. An example of this we find even among the heathen in Socrates, who calmly looking to the future receives the fatal cup.

From this point of view true old age is not the imperfect end, but the crown of humanity, in which it has ascended to true liberty, to the mastery over and direction of itself, and where only reason and understanding, moral feeling and piety hold the reins of government, while its vigour is softened by philanthropy; for love, the fairest flower of humanity, does not grow old with age.

Thus we see in this picture of our life, how the body is the vehicle and means of the development of our higher principle, and works harmoniously in the different periods, helping us and putting us in a state to attain to our appointed lot; the body grows old, but in the higher development of our mind we observe no retrogression.

After this rapid sketch of the development of the human mind, let us reflect for a moment on what has been said, and repeat once more the question, Does this picture teach us that soul and body are so completely one, that the soul is nothing but the unstable product of a material power, possessed of no independence? I wonder, in fact, at the power of those who can with such a conviction connect a belief in a future state. I do not possess this power; if all my reasons are snatched away from me, my belief no longer finds support. But does nature teach us this? By no means. If—I here repeat the antithesis—understanding and our moral feeling are nothing else than physical vital power and products of the metamorphosis of tissue, and not any proper independent subsistence, why are they so slight, indeed scarcely existent in the child, where everything in the body is full of life and action, and the metamorphosis of tissue is strongest; how is it possible that in the old man understanding, judgment, moral and religious feeling should have attained so high a pitch and become so highly developed, where the metamorphosis of tissue and all action and forces of the body exhibit so much less activity? Why in increased action of the body or brain, in passion and rage, is the action of the soul impeded and carried away? Why is it not rather as a product of exalted bodily actions itself increased? Why, if the soul possesses no substantial existence, does that which it has once made its own become its permanent property, which changes not with the altering play of its powers, nor diminishes in old age? Is it, in fact, not a singular contradiction, that we consider man to be independent and honour him as such, who offers resistance to and can suppress the passions and desires springing as impressions from his body, while we still deny independence to the high principle which places him in a condition to do this, endows him with the powers necessary to do it and raises him above these impressions? Is then, in fact, the soul nothing but the product of a material force, or, as Ludwig Fick* and others too, declare, nothing but the product of nervous currents? Then the effect works against the cause whence it arose, and even controls its power, which is to me inconceivable. Then the soul can be nothing else than a more or less excited vital force, and all moral responsibility is lost. Then it is a deception to suppose that nature has implanted in us an internal voice of conscience, proper to all men and nations; but not belonging to the animal creation. If we look at the old man—the innate sentiment of piety, which man could not learn from animals, occurs in him, purified from passions and impulses and developed to its fairest bloom, and with this innate feeling is connected a consciousness of continued existence in another world, which is implanted in all men. Would nature sport so cruelly with us, by implanting

* Müller's *Archiv.*, 1851, Heft V., page 385.

a lie in us? Is this the language of the Creator, which we read in his works? Can it be nervous force alone which exalts the human mind so high as to enable it not merely to determine the distance and movements of the heavenly bodies many hundred thousand millions of miles away, but even to weigh their masses and calculate their size?

But, it will be said, the natural philosopher admits only matter and material forces, which for him are one with matter, the immaterial in his opinion does not exist; all action proceeds from a material power united to matter. But who gives him a right to this position? Is there no action, unless combined with our coarse earthly matter, and does Nature herself exhibit herein no difference, no transition? Ask, then, the philosopher what matter the ether of light is, which he himself is constrained to assume, and the vibrations of which in a minute traverse many millions of miles? Fine as he may think this, it must, if it possesses the properties of our earthly matter, offer a certain amount of resistance to the upper layers of our atmosphere, which flies with our globe with more than the rapidity of a cannon ball through this ether, and impetuous currents of air and all devastating hurricanes must be the inevitable result; but this light-ether belongs not to our earth; it is a substance of the universe. Or, can we by the laws of ponderable matter, explain the fact, that a violent agitation of the magnet observed here takes place at the same moment in Asia and Siberia, in Europe and North America, and is at the same time accomplished at the South Pole in an opposite direction? Or is it in accordance with the phenomena of heavy matter, that the electric telegraph conveys our messages in a fraction of a second over a great portion of the globe?

It is, in my opinion, in a great measure the unfortunate distinction of material and immaterial which leads to so much confusion on this subject. Should we not pursue a safer course by distinguishing in Nature what is perceptible to our senses from that which is withdrawn from them? Who gives us the right to decide that the limits of Nature do not surpass the boundaries of our senses, and that no substances exist in her treasuries which we cannot perceive, measure, nor weigh? I will then rather consider our mind to be a substance beyond the reach of our senses, and withdrawn from the laws of terrestrial matter, than give up a belief which is inscribed in us by Nature herself. And if it is a position generally admitted by philosophers, that no matter, no substance, not even the smallest atom, disappears from creation, it follows that this exalted substance itself must be immortal.

But do we, in conclusion, find such properties in our soul? Not to speak of animal magnetism, of which all the phenomena cannot be denied, I shall venture to refer merely to two examples which occurred to myself, of two patients under my care at different times, one of whom assured me in the morning with great agitation, that he had by some inexplicable perception learned the death of his father, the other of her husband; neither knew anything of the illness of the party concerned, and three days later I received from a distant province the account of the death of the father, and in the other case, on the following day, from an adjoining town, of that of the husband, each event having occurred at the very moment of the perception. Although with respect to such statements, I must most earnestly warn my readers against credulity and even superstition,—for which reason I have always made it a rule, having had many such circumstances communicated to me by credible persons, not indeed to deny them, but to depend only on what I had myself clearly observed—it appears to me more difficult to attribute these, and several similar cases which have come under my knowledge, to mere accident, than to believe that, under some extraordinary circumstances, our spirit may enter into connection with hidden powers in Nature, thus manifesting a capacity above time and space, which is certainly not planted in our soul for this earthly abode.

Herder correctly observes:* "Some examples of recollection, of the power of imagination, and even of prescience, have revealed wonders of the concealed treasures which slumber in the human soul, but cannot develope themselves there; and to Him, who placed so many powers in the body, and planted the soul above them, assigned them a working-place and fixed the nerves as the paths whereby the soul may work on these powers, the means shall not be wanting, in the great system of nature, of again eliciting those which he has so wonderfully and distinctly placed for higher development in this organic dwelling."

---

### Art. IV.—NOTES ON THE ASYLUMS OF ITALY, FRANCE, AND GERMANY.

**Philosophie der Geschichte*, 1 Th., pages 193 and 163.

